# Remote synchronous usability testing of public access defibrillators during social distancing in a pandemic

**DOI:** 10.1038/s41598-022-18873-7

**Published:** 2022-08-26

**Authors:** Hannah Currie, Adam Harvey, Raymond Bond, Justin Magee, Dewar Finlay

**Affiliations:** 1 Ulster University, Jordanstown, UK; 2 HeartSine Technologies Ltd., Belfast, UK; 3 Stryker, Belfast, UK

**Keywords:** Biomedical engineering, Cardiac device therapy

## Abstract

Public access automated external defibrillators (AEDs) represent emergency medical devices that may be used by untrained lay-persons in a life-critical event. As such their usability must be confirmed through simulation testing. In 2020 the novel coronavirus caused a global pandemic. In order to reduce the spread of the virus, many restrictions such as social distancing and travel bans were enforced. Usability testing of AEDs is typically conducted in-person, but due to these restrictions, other usability solutions must be investigated. Two studies were conducted, each with 18 participants: (1) an in-person usability study of an AED conducted in an office space, and (2) a synchronous remote usability study of the same AED conducted using video conferencing software. Key metrics associated with AED use, such as time to turn on, time to place pads and time to deliver a shock, were assessed in both studies. There was no difference in time taken to turn the AED on in the in-person study compared to the remote study, but the time to place electrode pads and to deliver a shock were significantly lower in the in-person study than in the remote study. Overall, the results of this study indicate that remote user testing of public access defibrillators may be appropriate in formative usability studies for determining understanding of the user interface.

## Introduction

Out-of-hospital cardiac arrest (OHCA) accounts for 300,000 and 420,000 deaths per year in Europe and the US respectively^1,2^. Survival of sudden cardiac arrest decreases with time when the patient remains untreated^3^. If a defibrillating shock is administered within 3 minutes, the chances of survival to hospital discharge increase by up to 74%^4^. Public access automated external defibrillators (AEDs) are medical devices which are often used by untrained or minimally trained laypersons to deliver a shock to victims of OHCA. Successful treatment of a patient with an AED relies on the completion of four key tasks: (1) turning on the device, (2) correctly placing the self-adhesive electrode pads which enable recording of the electrocardiogram, (3) removing contact with the patient during analysis/shock, and (4) enabling shock delivery. It is vital that public access AEDs are intuitive to use and are designed to mitigate human error, as incorrect execution of any of these key tasks could cause delayed or suboptimal treatment, such as reduced efficacy of defibrillating shocks due to inaccurate pad placement, which may impact survival outcomes^5^.

To ensure that public access AEDs are used as intended and that their usability is optimized, simulation testing with a representative sample of users is a regulatory requirement to be fulfilled before approving the devices for sale^6–8^. It is advised that the usability testing of medical devices is iterative, with usability evaluations occurring early and often in the design and development process providing many ‘formative’ assessments leading to a ‘summative’ evaluation of the final device^9^. However, the current environment has challenged usability testing processes given that this task typically requires close human contact with members of the public.

In 2020, the novel coronavirus, SARS-COV-2 (commonly known as Covid-19) caused a global pandemic, infecting over 35 million people as of November 2020^10^. In order to minimise the spread of Covid-19, many restrictions were declared worldwide in early 2020. Population movement was restricted in 186 countries, both locally and internationally^11–13^. The US Centers for Disease Control and Prevention and the US government announced restrictions on international travel into the US^14,15^. The United Kingdom announced significant social restrictions in March 2020, including advising the public to maintain at least a 2-m distance from others and work from home unless the work is “absolutely necessary”^16^. Similarly, the World Health Organization recommended that meetings in indoor settings are avoided, and that persons maintain a minimum of 1-m distance from others^17^. The impact of Covid-19 has meant that social interaction, and in-person usability simulation testing is not possible without risk to the test participants and investigators. During this time, a larger proportion of business and social interactions occur remotely, utilizing video conferencing technology and interactive tools. In line with this, alternative methods to assess usability and understanding of user interactions with medical devices such as public access defibrillators must be developed.

Remote usability testing has been widely used in computing to test mobile phones, websites and applications. Methodology varies in terms of tools used, with some groups utilizing publicly available conferencing and screen-sharing software or proprietary software tools^18–20^, but also in terms of observation. Remote user testing can be described as synchronous, where the testing is conducted in real-time with an observer^21^, or asynchronous, where the testing is not conducted in real-time without observation, for example, questionnaires delivered by email^19^. Synchronous and asynchronous testing have been shown to provide similar results when assessing the usability of a website^21^, but asynchronous has less observer involvement which may impact any post-test discussions^22^. Sun and Hewlett Packard reported conducting remote user testing through video conferencing in 1994, stating that visual cues helped establish rapport with participants which enabled more valuable discussions and results^22^. More recently, virtual reality has been used to increase the sense of realness of remote user testing^23^.

Remote usability testing of medical devices is less common. The increased demand and utilization of home-healthcare equipment, and the use of medical devices in the home, prompted usability assessment of selected devices in an asynchronous remote study. Kortum and Peres measured the usability of 23 home health care devices, including thermometers, blood pressure cuffs and inhalers, using the System Usability Scale^24,25^. Public access defibrillators were not considered in this study by Kortum and Peres. Indeed, remote usability testing of public access defibrillation has not yet been reported.

The objective of this study was to assess if remote usability testing through video conferencing programs is a suitable method for conducting formative usability evaluation of public access defibrillators. In addition, we aim to assess if the results of remote usability testing are comparable to in-person simulation testing of the same device.

## Methods

**Figure 1:**
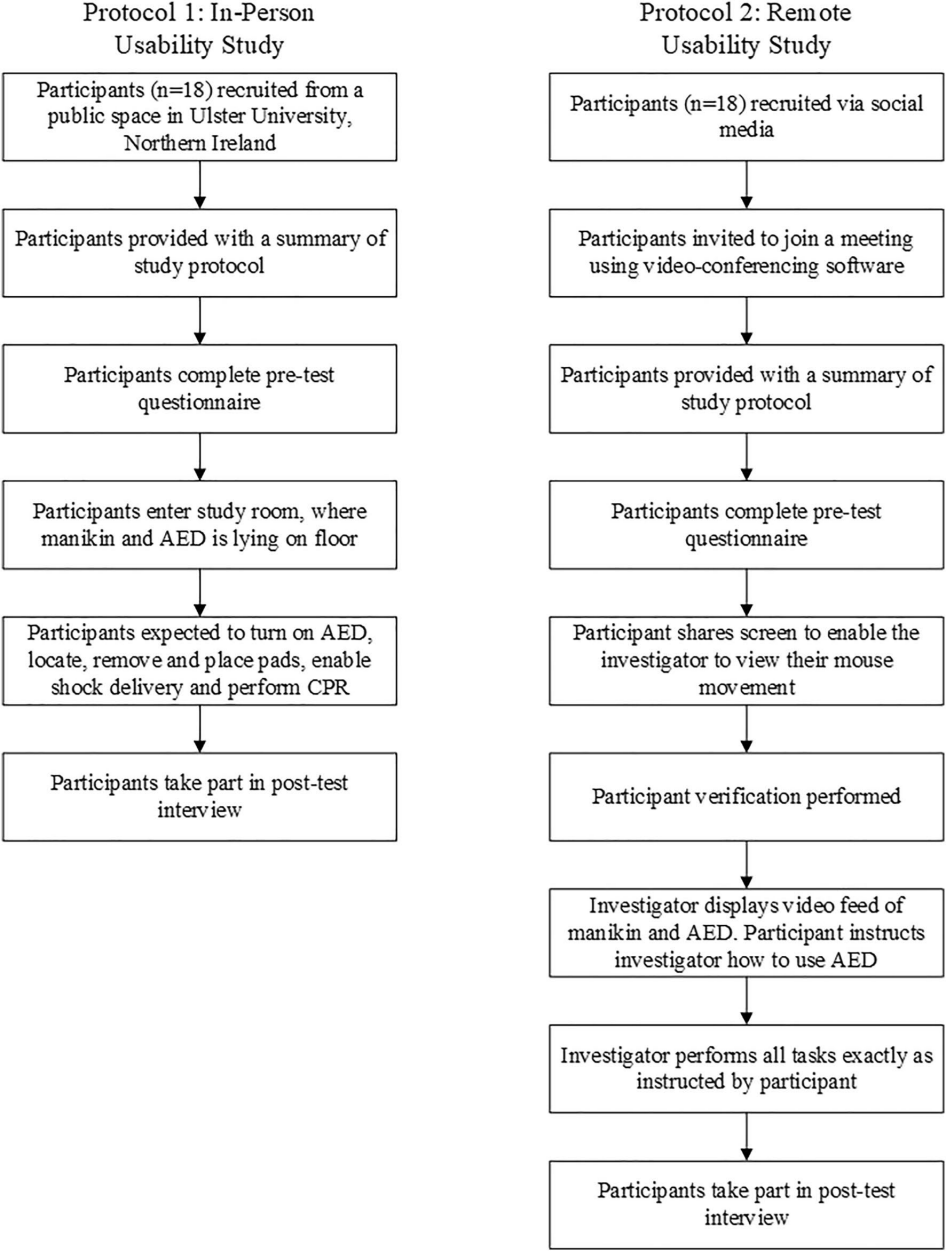
A flow chart depicting the study protocols for the in-person and remote usability studies.

This study was reviewed and approved by the Ulster University Faculty of Computing, Engineering and Build Environment Ethics Committee (Reference: 15.07 Bond, Raymond [Torney, Hannah]). The study was executed in two parts: Protocol 1 was an in-person usability study of the HeartSine SAM 500P (HeartSine, Belfast, Northern Ireland), and Protocol 2 was a remote usability study of the same device, conducted using video-conferencing software. Sample sizes for each protocol were based on usability and human factors guidance, which recommend a minimum of 15 participants per group^7,9^. Figure 1 provides a flow-chart depicting each study protocol.

### Protocol 1: In-person usability study

The simulation study was executed in an office space in Ulster University, Northern Ireland in January 2017, prior to the Covid-19 pandemic. Eighteen participants were randomly recruited from a public space in Ulster University and provided informed consent. Parental informed consent was gained for participants aged less than 18 years.

### Equipment set-up

**Figure 2:**
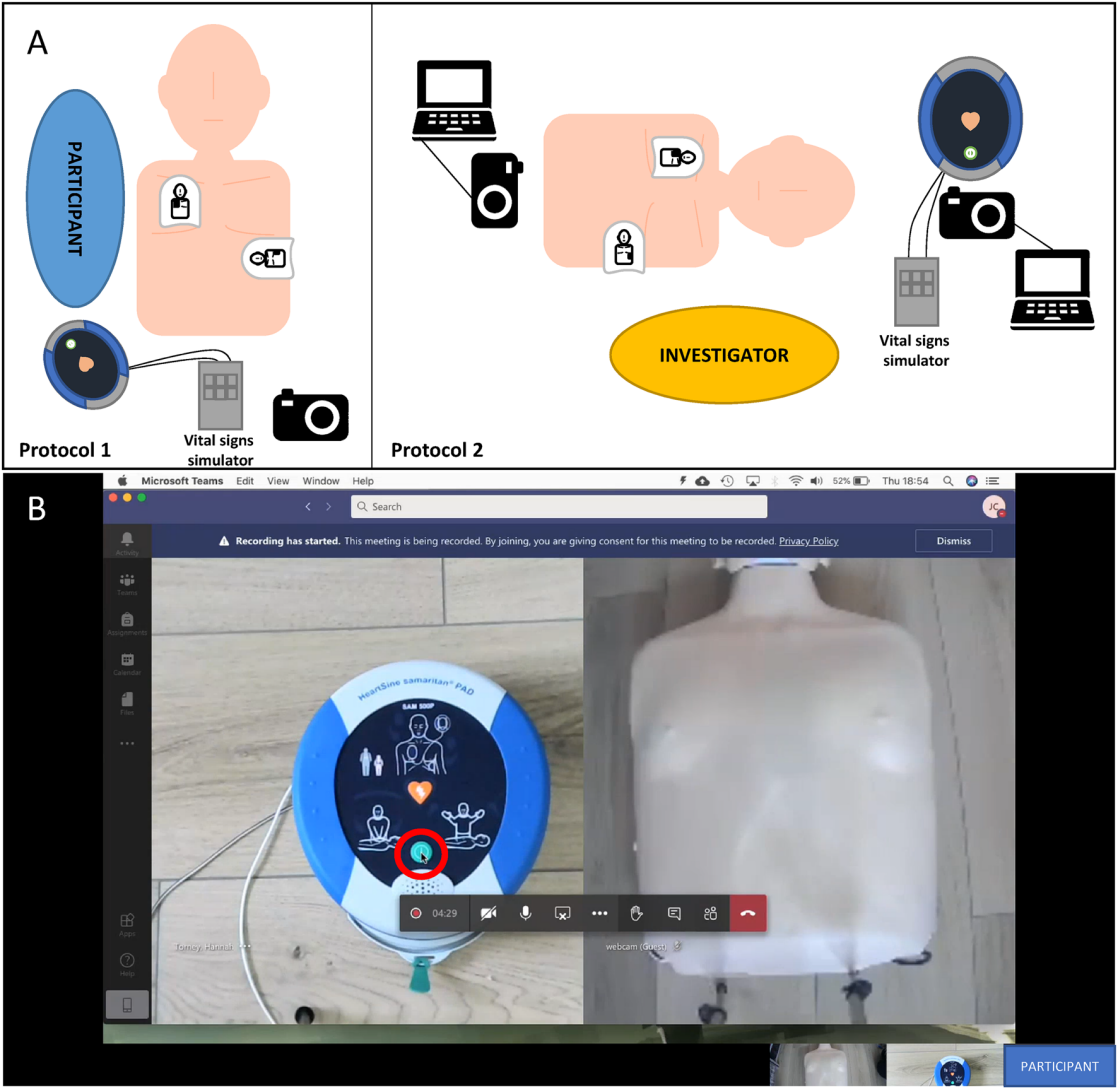
(**A**) Equipment set-up for Protocols 1 and 2. In Protocol 1, the AED was connected to the vital-signs manikin ECG simulator through the electrode cables. A video camera was placed on a tripod ensuring clear view of the equipment and participant. In Protocol 2, the SAM 500P was connected to the vital-signs manikin ECG simulator through the electrode cables. A webcam connected to a laptop with Microsoft Teams provided a view of the AED. A second webcam and laptop with Microsoft Teams provided a view of the vital-signs manikin. The investigator was located between the manikin and the AED to perform tasks as instructed by the participant. (**B**) The plane-view of the AED and the manikin are visible as side-by-side video feeds. The participant has shared their screen, enabling the investigator to view their mouse movements over the video feed (see mouse circled). The participant’s video icon on the bottom right of the screen has been anonymized.

This study assessed the in-person usability of the HeartSine SAM 500P. To ensure the safety of the participants and investigators, the AED used throughout the study was modified to remove hardware which prevents the device from physically delivering a shock. Otherwise, the device was fully representative of the marketed SAM 500P. A vital signs manikin (TruCorp, Craigavon, Northern Ireland) capable of simulating shockable arrhythmias was placed on the floor with the SAM 500P beside it. The equipment set up for Protocol 1 is shown in Fig. 2.

### Test phase

Upon recruitment, the participants were provided with a summary of the study protocol outlining basic background information regarding the study. All participants were assigned an identification number and requested to complete a pre-test questionnaire which collected the participants’ demographics and if they had previous cardiopulmonary resuscitation (CPR) or defibrillation training. In addition, participants were asked to score how difficult they expected the test to be, on a scale of 1–10, where 1 was very easy and 10 very difficult.

The participants were instructed to enter the test room where they found a simulated cardiac arrest victim (the clothed manikin described above) lying on the floor. They were instructed to assume emergency services had already been called and that the victim was not breathing and did not have a pulse. They were then told they would find an Automatic External Defibrillator, or AED next to the victim which could be used for the delivery of an electrical shock to a patient’s heart to restart it pumping again. Participants were informed that one or more persons would be observing and video recording their actions; but they may not ask them questions or ask for help until after they had been advised the test was complete. They were told that they should act in the same manner as they might during an actual emergency where time is critical and every second counts. The participants were reassured that it was the AED that was being evaluated, not them, but that it was important that they act with the same sense of urgency, determination, and care that they would bring to a real emergency situation of this kind. The investigator indicated the start of the test by saying “you may begin”. This instruction indicated the start time for any time-based metrics in the study. Each test was conducted in a room without interruption and the participant was video recorded for the duration of the testing to allow for retrospective analysis.

Participants were expected to turn on the AED, locate and remove the pads from the AED, remove the plastic liner and place the pads on the manikin, remove contact with the manikin when instructed by the device, enable shock delivery and perform a two-minute cycle of CPR.

Upon completion, the participants were asked to score the actual difficulty of the test, on a scale from 1 to 10, where 1 was very easy and 10 very difficult. They were then invited to take part in a short interview to provide information on their opinion of the test and the usability of the device.

### Protocol 2: remote usability study

This simulation study was conducted remotely using video conferencing software in September 2020. Eighteen participants were recruited after responding to a social media post and informed consent was obtained. There were no participants aged less than 18 years in this study.

### Equipment set-up

To enable comparison to Protocol 1, this study also assessed the usability of the HeartSine SAM 500P. To ensure the safety of the investigator, the SAM 500P used throughout the testing was modified in the same way as Protocol 1. Otherwise, the device was fully representative of the marketed SAM 500P. A webcam connected to Microsoft Teams (Redmond, USA) was placed over the AED providing plane view of the device. A vital signs manikin (TruCorp, Craigavon, Northern Ireland) was placed on the floor, also with a webcam connected to Microsoft Teams in plane-view as demonstrated in Fig. 2. Participants were invited to join individual Microsoft Teams meetings. Two participants were unable to join the Microsoft Teams meeting, and Google Meet was used instead. There were no differences in study set up between Microsoft Teams and Google Meet.

The participants were given a brief outline of the study, which gives information on AEDs and their use. All participants were assigned an identification number and requested to complete a Pre-Test Questionnaire which was identical to that used in Protocol 1. The participants were also asked to score how difficulty they expected the test to be, on a scale from 1 to 10, where 1 was very easy and 10 very difficult.

Participants were informed that the study was being conducted to assess the usability of the device and feasibility of remote usability testing. They were informed that they would be asked to instruct the investigator to use a public access automated external defibrillator, providing instruction both verbally and visually through movement of their computer mouse over a video feed. The investigator instructed the participant to share their screen, allowing the participant to indicate their movements with their mouse.

### Participant verification

Before commencing the test phase, a two-step participant verification was executed to assess video latency and provide an example scenario. Firstly, the investigator placed a sheet of paper with the letters A and B written on it under the webcam view and instructed the participant to move their mouse between the letters, stating which they are indicating as they moved their mouse. This indicated any latency in the video display. Secondly, the investigator placed a television remote control under the webcam view and asked the participant to visually (with their computer mouse) and verbally guide the investigator to turn on the television and open a particular application. This enabled the participant to practice how to instruct the investigator.

### Test phase

Prior to beginning the study, the investigator informed the participant that they had come across a person who was suffering sudden cardiac arrest. They were told that 999 had been called, and that the investigator had a public access AED, but that they did not know how to use it and they needed their guidance. They were told that sudden cardiac arrest is time-critical and that they needed to think quickly and provide clear instruction. The investigator indicated the start of the test by saying “Let’s begin”. This instruction indicated the start time for any time-based metrics in the study.

The investigator performed all tasks as instructed and indicated. For example, a participant may instruct the user “Press the button on the middle of the device”. Importantly, to avoid bias, the investigator did not make any assumptions, instead clarifying with the participant and requesting further instruction, such as “Which button do you want me to press?”.

Similar to Protocol 1, participants were expected to instruct the investigator to turn on the AED, locate and remove the pads from the AED, remove the plastic liner and place the pads on the manikin, remove contact with the manikin when instructed by the device and enable shock delivery. Upon completion, participants were asked to score the actual difficulty of the test and they partook in a short interview to provide information on their opinion of the test protocol and the usability of the device.

### Study metrics

The key metrics in this study were the time taken to turn on the AED, time taken to place the pads and time taken to deliver a shock. Acceptability of the pad placement was assessed by considering the quadrant of the thorax on which the pads were placed. Sternal pad placement was considered correct if located between the collarbone and nipple, to the right of the sternum, and the apical pad placement was considered correct if positioned below the left nipple, close to the left mid-axillary line. In addition, safety was assessed through the proportion of participants who did not touch the manikin during the analysis period of the algorithm in Protocol 1, and those who indicated that the investigator should “stand clear” in Protocol 2Of note, both Protocol 1 and Protocol 2 used the same investigator who, beyond introducing the scenario, did not provide guidance in either study, but sought clarification in the remote study in Protocol 2 if the instructions were not clear.

### Statistical analysis

Minitab 19 was used for data analysis. Summary statistics were calculated for participant age and gender, education level and previous CPR and/or defibrillation training. Pearson’s χ^2^ test and Mann–Whitney U tests were used where appropriate. A p value of < 0.05 was considered statistically significant.

## Results

Eighteen participants were recruited to each protocol. No same participant was recruited for both protocols. All methods were carried out in accordance with the study protocols, and where appropriate, Covid-19 guidelines and regulations. The participant demographics are reported in Table 1.

**Table 1:** Demographic information for participants enrolled in Protocol 1: In-Person Study and Protocol 2: Remote Study.

	Protocol 1: in-person (n = 18)	Protocol 2: remote (n = 18)	*p*
**Native language**			
English	18 (100%)	17 (94.4%)	N/A
Other	0 (0%)	1 (5.6%)	
**Age**			
Median age	22 years	27 years	0.255
15–20	4 (22.2%)	1 (5.6%)	
21–30	10 (55.6%)	13 (72.2%)	
31–40	1 (5.6%)	1 (5.6%)	
41–50	3 (16.7%)	0 (0.0%)	
51–60	0 (0%)	3 (16.7%)	
**Gender**			
Male	14 (77.8%)	9 (50.0%)	0.08
Female	4 (22.2%)	9 (50.0%)	
**Education**			
No College, University or Higher	6 (33.3%)	6 (33.3%)	0.903
College, University or Higher	11 (61.1%)	12 (66.6%)	
Unreported	1 (5.6%)	0 (0.0%)	
**Previous experience**			
CPR	9 (50.0%)	11 (61.1%)	0.502
Defibrillation	5 (27.8%)	7 (38.9%)	0.480
Neither CPR nor defibrillation	9 (50.0%)	7 (38.9%)	0.502

**Figure 3:**
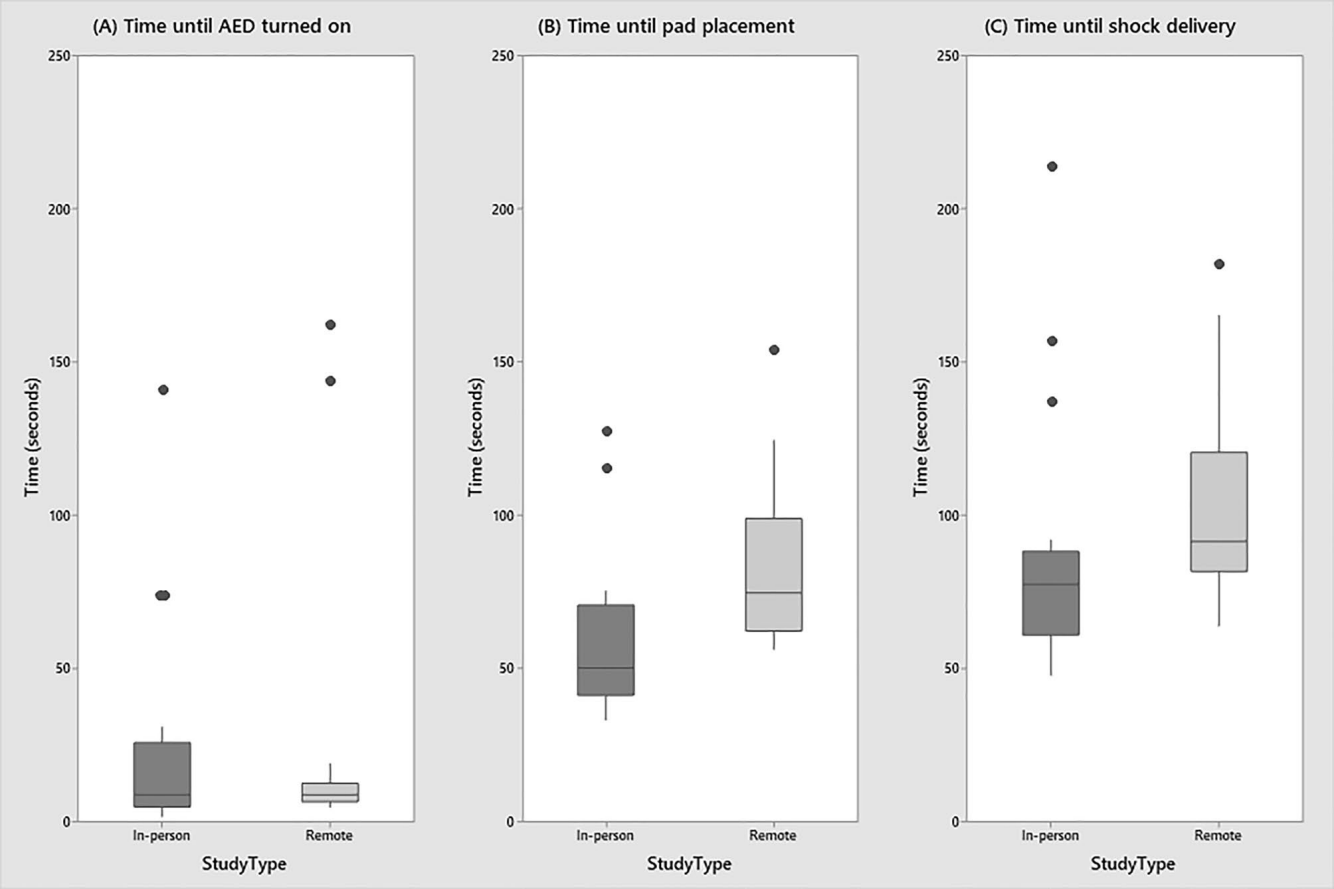
Box plots depicting (**A**) the time taken to turn the AED on, (**B**) time until pad placement and (**C**) deliver a shock in Protocol 1: In-person study and Protocol 2: Remote study.

The descriptive statistics relating to time take to turn on the AED, place the pads and deliver a shock are reported in Table 2 and Fig. 3.

**Table 2:** Descriptive statistics for time taken to turn on AED, place the pads and deliver a shock, acceptable electrode placement and safety metrics in both Protocol 1: In-Person study and Protocol 2: Remote study. *Denotes statistical significance.

	Protocol 1: in-person (n = 18)	Protocol 2: remote (n = 18)	*p*
Time to turn on AED (median (IQR), s)	9.0 (5.0, 26.0)	9.0 (6.8, 12.8)	0.830
Time to place pads (median (IQR), s)	50.0 (41.0, 70.5)	73.5 (64.3, 98.8)	< 0.01*
Time to deliver shock (median (IQR), s)	77.5 (61.0, 88.3)	91.5 (83.5, 120.5)	< 0.05*
AED on to Pads on interval (median (IQR), s)	41.0 (35.0, 49.0)	65.0 (55.3, 73.0)	< 0.001*
Pads on to Shock interval (median (IQR), s)	20.0 (16.5, 23.3)	19.0 (17.8, 20.0)	0.681
Participants (n, %) with acceptable electrode placement	18 (100%)	14 (77.8%)	< 0.05*
Participants (n, %) who did not make contact during first analysis	15 (83.3%)	N/A	N/A
Participants (n, %) who instructed investigator to not make contact during first analysis	N/A	7 (38.9%)	N/A

While there was no difference in the time taken to the turn on the AED in the in-person study versus remote study [9.0 s (5.0, 26.0) vs 9.0 s (6.8, 12.8)], the time taken to place the pads was lower in the in-person study testing [50.0 s (41.0, 70.5) vs 73.5 s (64.3, 98.8)]. This translated to a faster time taken to deliver a shock in the in-person study [77.5 s (61.0, 88.3) vs. 91.5 s (83.5, 120.5)].

It was expected that users would turn the AED on before placing the pads. However, we observed that 3 (16.7%) participants in Protocol 1, and 2 (11.1%) in Protocol 2 removed and placed the pads before turning on the AED, and of these participants, the mean (SD) time taken to place the pads was 75.3 (44.8) seconds in Protocol 1, and 137.0 (24.0) seconds in Protocol 2 (p = 0.185). All participants (100%) in Protocol 1 and 14 (77.8%) participants in Protocol 2, correctly placed the electrodes as indicated by the icons on the SAM 500P user interface and the pads.

To compare how difficult participants of both studies expected the tasks to be and how difficult they actually found them, they were asked to rate perceived difficulty (1–10) prior to commencing the tasks and actual difficulty (1–10) after completion. The mean (SD) scores of perceived and actual difficulty are presented in Table 3.

**Table 3:** Descriptive statistics for scores of pre-conceived and actual difficulty in both Protocol 1: In-Person study and Protocol 2: Remote study. *Denotes statistical significance.

	Protocol 1: in-person (n = 18)	Protocol 2: remote (n = 18)	*p*
**Pre-conceived difficulty**			
Mean (SD)	5.4 (1.6)	4.9 (1.9)	0.403
**Actual difficulty**			
Mean (SD)	3.8 (2.2)	3.2 (2.4)	0.448
*p*	< 0.05*	< 0.05*	

## Discussion

This study was designed to compare in-person and remote simulation testing of a public access AED in order to understand the utility of remote user testing of medical devices. The fundamental actions required to successfully use an AED; place the pads and deliver a shock were compared between investigational approaches. The key findings of this study were that there was no statistical difference in time taken to turn on the AED (p = 0.830) between protocols. Participants of the in-person simulation study were more efficient in placing the pads (p < 0.01) and were more likely to achieve acceptable pad placement (p = 0.03). The time taken to deliver a shock was faster in the in-person simulation (p = 0.014). In both studies, participants found the test easier than they initially expected [perceived vs. actual difficulty: Protocol 1: 5.4 (1.6) vs 3.8 (2.2), p = 0018; Protocol 2: 4.9 (1.9) vs. 3.2 (2.4), p = 0.029], however, there was no difference in difficulty scores between Protocol 1 and 2.

This remote usability protocol was a synchronous test, where the participant interacted with the AED and the investigator in real-time. Previous studies have compared task completion time in lab-based and synchronous remote usability studies of websites. Higher task completion times in synchronous remote testing have been observed when compared to lab-based testing^20,26^, with other studies reporting no difference in task completion time^21^. Our study reports a similar time taken to turn on the AED, suggesting that the power button was easy to locate on the interface in both protocols. However, the task completion times diverge after the AED is switched on. Time taken to place the pads was significantly longer (on average longer by 23.5 s) in the remote study than the in-person study. The pads used in this study were the typical reusable variety used in training scenarios, without protruding wires. They were adhered to a transparent plastic liner which must be removed before placing on the manikin. Four (22.2%) participants in the remote study did not initially instruct the investigator to remove the liner before placing the pads on the manikin. The median (IQR) time to place the pads for these participants was 102.5 (85.3, 120.5) seconds. Three of these participants identified the pad placement as the hardest part of the test when questioned in the post-test interview. It is possible that the transparent plastic liner was not clearly visible on the video recordings and removing the plastic liner is more intuitive in the in-person study. This increased time pad placement affected the time taken to deliver a shock which was also significantly longer in the remote study when compared to the in-person study. However, the interval between pad placement and shock delivery showed little difference between the study groups. This interval includes the ECG analysis time, after which the defibrillator will indicate whether a shock is or is not required. The analysis time was the same in both studies. The similar pads-to-shock interval may therefore indicate that the participants were able to locate the shock button easily in both studies.

Participants were more hesitant to begin in the remote study and 7 participants needed a prompt to begin, as although the final instruction from the investigator was “let’s begin”, they did not appear to realise that this was the beginning of the test. This may suggest lower sense of urgency in the remote study or may highlight an effect of the different wording between the studies (Protocol 1: “You may begin”, Protocol 2: “Let’s begin”). Considering the similarity in perceived vs. actual difficulty scores (Table 3), it may be the case that the technical aspect of the remote usability testing (i.e., sitting in front of a computer, with a calm environment shown on video feed) does not add undue stress or difficulty for the participants, hence lowering the fidelity of the simulated emergency.

During the ECG analyses, the device emits audio and visual instruction to “stand clear of patient, do not touch the patient”. In the in-person study, 15 of the 18 (83.3%) participants did not make contact with the manikin during this time while 7 of the 18 (38.9%) participants instructed the investigator to stand clear of the patient during the remote study. This may have been impacted by the layout of the equipment, where the participants did not see a view of the AED, manikin and investigator in one frame. In addition, there were no occurrences where the investigator was touching the manikin during the analysis mode and the participant needed to instruct them to remove contact. Of note, both Protocol 1 and Protocol 2 used the same investigator to reduce variability and bias.

With technologies such as virtual and augmented reality, there are possibilities for development of novel usability testing protocols which could be conducted through asynchronous remote testing. Madathil and Greenstein reported the use of a virtual reality system to conduct moderated usability testing^23^ and Ingrassia et al. recently reported the potential use of augmented reality in moderated basic life support and defibrillation training^27^. However, similar systems could be used without moderation. Resuscitation Council (UK) developed an immersive serious game-style CPR training, Lifesaver (https://www.life-saver.org.uk/). Researchers compared CPR quality metrics when resuscitation training was provided using Lifesaver compared to face-to-face training^28^.

The authors noted however that there were benefits to conducting both face-to-face training and Lifesaver training, and perhaps this approach could be adopted in the usability testing of public access defibrillators. Virtual reality or serious games could be used for asynchronous remote usability testing early in the design process (formative testing), with an in-person face-to-face summative study on the final product. This could enable larger formative studies, which could provide greater insight into understanding of the user interface and audio-visual prompts, with less burden on the investigators.

This study was not statistically powered and the sample sizes for both protocols were based on usability and human factors guidance and standards, which recommend a minimum of 15 participants per group^7,9^. Comparison between demographic groups was therefore not appropriate due to small group sizes. Additional participants could be recruited in order to compare demographic groups such as gender or education level between remote and in-person protocols.

The participants recruited during both protocols were reasonably young (Median, Protocol 1: 22 years, Protocol 2: 27 years). Future studies could consider the recruitment of older participants, who may behave differently in both protocols. Similarly, Protocol 1 had an uneven split between genders, which could be considered in future studies.

A key aspect of resuscitation and public access AED use is the participant’s ability to follow instructions to perform CPR. CPR performance is commonly measured through analysis of compression rate, depth and fraction, and performance can depend on the individual rescuers. For example, rescuer weight can impact chest compression depth^29,30^. Thus, a limitation of this study was that the participant’s CPR performance could not be analysed through this study design. This could be considered during future work. In addition, CPR is known to be physically intensive, so future work could include physiological measurements to detect and measure stress during the scenarios.

A final limitation to this study is the length of time between the conduct of Protocol 1: In-person study and Protocol 2: Remote. Protocol 1 was conducted in January 2017 and Protocol 2 was conducted in September 2020. Since 2017, it is possible that there may be an increased awareness of public access defibrillation and thus the study populations may be slightly different. However, there was no statistical difference between the study groups in terms of previous CPR or defibrillation experience (Table 1).

CPR training with virtual reality technologies has recently been reported and whilst this study outlined a method for assessing usability of a public access AED, the same method could be appropriate for training lay-users^31–33^. At the time of this study, this was the only possible way to conduct human factors testing due to the pandemic and may represent a feasible early phase method for testing medical devices and providing CPR training beyond the pandemic.

## Conclusion

This study is the first of its kind comparing the usability of a public access defibrillator in in-person and remote simulation studies. Overall, the results of this study indicate that remote user testing of public access defibrillator may be appropriate in formative usability studies for determining understanding of the user interface, as indicated by the rapid time taken to turn on the device, and similar pads-to-shock interval. It should be noted however, that most time-based metrics may not be reflective of actual use, and an in-person summative study remains necessary.

## References

[1] Atwood C, Eisenberg MS, Herlitz J, Rea TD (2005). Incidence of EMS-treated out-of-hospital cardiac arrest in Europe. Resuscitation.

[2] Go AS (2014). Heart disease and stroke statistics-2014 update: A report from the American Heart Association. Circulation.

[3] Larsen MP, Eisenberg MS, Cummins RO, Hallstrom AP (1993). Predicting survival from out-of-hospital cardiac arrest: A graphic model. Ann. Emerg. Med..

[4] Valenzuela TD, Roe DJ, Cretin S, Spaite DW, Larsen MP (1997). Estimating effectiveness of cardiac arrest interventions: A logistic regression survival model. Circulation.

[5] Esibov A, Chapman FW, Melnick SB, Sullivan JL, Walcott GP (2016). Minor variations in electrode pad placement impact defibrillation success. Prehospital Emerg. Care.

[6] E. Commission. Regulation (EU) 2017/745 of the European Parliament and of the Council of 5 April 2017 on medical devices, amending Directive 2001/83/EC, Regulation (EC) No 178/2002 and Regulation (EC) No 1223/2009 and repealing Council Directives 90/385/EEC and 93/42/EE. (2017).

[7] FDA and U.S. Department of Health and Human Services. Applying Human Factors and Usability Engineering to Optimize Medical Device Design (2016).

[8] Torney H (2016). A usability study of a critical man-machine interface: Can layperson responders perform optimal compression rates when using a public access defibrillator with automated real-time feedback during cardiopulmonary resuscitation?. IEEE Trans. Hum. Mach. Syst..

[9] American National Standard. *ANSI/AAMI/IEC 62366-1:2015 Medical Devices—Part 1: Application of Usability Engineering to Medical Devices* (American National Standard, 2015).

[10] Alwan, N. A. *et al.* Scientific consensus on the COVID-19 pandemic: We need to act now. *Lancet*. 71–72 (2020). 10.1016/S0140-6736(20)32153-X PMC7557300 33069277

[11] Devi S (2020). Travel restrictions hampering COVID-19 response. Lancet.

[12] E. Han *et al.* Lessons learnt from easing COVID-19 restrictions: An analysis of countries and regions in Asia Pacific and Europe. *Lancet*. 1525–1534 (2020). 10.1016/S0140-6736(20)32007-9 PMC7515628 32979936

[13] UNICEF. Don’t let children be the hidden victims of COVID-19 pandemic: Statement by UNICEF Executive Director Henrietta Fore [Internet]. *UNICEF.* (2020).

[14] Trump, D. J. *Suspension of Entry as Immigrants and Nonimmigrants of Certain Additional Persons Who Pose a Risk of Transmitting 2019 Novel Coronavirus* (United States Government, 2020).

[15] Centers for Disease Control and Prevention. Travelers Prohibited from Entry to the United States. *CDC*, 2020. [Online]. https://www.cdc.gov/coronavirus/2019-ncov/travelers/from-other-countries.html.

[16] Johnson, B. Prime Minister’s Statement on Coronavirus (COVID-19): 16 March 2020’. *GOV. UK*, vol. 16 (2020).

[17] World Health Organization. Considerations in adjusting public health and social measures in the context of COVID-19. *World Heal. Organ. Interim Guid.*, April, 1–7 (2020).

[18] Scholtz, J. Adaptation of traditional usability testing methods for remote testing. In *Proc. 34th Hawaii Int. Conf. Syst. Sci.* 1–9 (2001).

[19] Martin, R., Al Shamari, M., Seliaman, M. E., & Mayhew, P. Remote asynchronous testing: A cost-effective alternative for website usability evaluation. *Int. J. Comput. Inf. Technol.* 2279–0764 (2014).

[20] Andrzejczak C, Liu D (2010). The effect of testing location on usability testing performance, participant stress levels, and subjective testing experience. J. Syst. Softw..

[21] Sauer J, Sonderegger A, Heyden K, Biller J, Klotz J, Uebelbacher A (2019). Extra-laboratorial usability tests: An empirical comparison of remote and classical field testing with lab testing. Appl. Ergon..

[22] Hammontree M, Weiler P, Nayak N (1994). Remote usability testing. Interactions.

[23] Chalil K, Greenstein JS (2017). An investigation of the efficacy of collaborative virtual reality systems for moderated remote usability testing. Appl. Ergon..

[24] Kortum P, Peres SC (2015). Evaluation of home health care devices: Remote usability assessment. JMIR Hum. Factors.

[25] Bangor A, Kortum PT, Miller JT (2008). An empirical evaluation of the system usability scale. Int. J. Hum. Comput. Interact..

[26] Thompson, K. E., Rozanski, E. P., & Haake, A. R. Here, there, anywhere : remote usability testing that works. In *Proc. 5th Conf. Inf. Technol. Educ.*, 132–137 (2004).

[27] Ingrassia PL (2020). Augmented reality learning environment for basic life support and defibrillation training: Usability study. J. Med. Internet Res..

[28] Yeung J (2017). The school Lifesavers study—A randomised controlled trial comparing the impact of Lifesaver only, face-to-face training only, and Lifesaver with face-to-face training on CPR knowledge, skills and attitudes in UK school children. Resuscitation.

[29] Hasegawa T, Daikoku R, Saito S, Saito Y (2014). Relationship between weight of rescuer and quality of chest compression during cardiopulmonary resuscitation. J. Physiol. Anthropol..

[30] Cornara S (2015). How weight and other anthropometric variables affect CPR quality: A study on lay rescuers. Resuscitation.

[31] Lee S, Hong K, Choi S (2022). Usability, acceptability, and feasibility of an online, real-time home CPR training solution (HEROS-remote) during the COVID-19 pandemic. BMJ Open.

[32] Leary M, McGovern SK, Chaudhary Z, Patel J, Abella BS, Blewer AL (2019). Comparing bystander response to a sudden cardiac arrest using a virtual reality CPR training mobile app versus a standard CPR training mobile app. Resuscitation.

[33] Moll-Khosrawi P, Falb A, Pinnschmidt H, Zöllner C, Issleib M (2022). Virtual reality as a teaching method for resuscitation training in undergraduate first year medical students during COVID-19 pandemic: A randomised controlled trial. BMC Med. Educ..

